# Economic Evaluation of Multi-Objective Schistosomiasis Control Through Systemic Causality: Theoretical Advances and Governance Implications

**DOI:** 10.3390/tropicalmed11030072

**Published:** 2026-03-05

**Authors:** Menghua Yu, Xinyue Liu, Na Shi, Jiaqi Su, Lefei Han, Jian He, Yaoqian Wang, Suying Guo, Wangping Deng, Chao Lv, Lijuan Zhang, Bo Fu, Hanhui Hu, Jing Xu, Xiao-Nong Zhou, Xiaoxi Zhang

**Affiliations:** 1 School of Global Health, Chinese Center for Tropical Diseases Research, Shanghai Jiao Tong University School of Medicine, Shanghai 200025, China; yumenghua@sjtu.edu.cn (M.Y.); lfhan@sjtu.edu.cn (L.H.); hj5566@126.com (J.H.); 2 School of Public Health, Shanghai Jiao Tong University School of Medicine, Shanghai 200025, China; 3 School of Health Sciences, Faculty of Medicine and Health, University of New South Wales, Sydney 2031, NSW, Australia; xinyue.liu11@unsw.edu.au; 4 West China School of Public Health and West China Fourth Hospital, Sichuan University, Chengdu 610041, China; bananana0225@163.com; 5 Asian Infrastructure Investment Bank (AIIB), Beijing 100101, China; jiaqi.su@aiib.org; 6 Key Laboratory on Technology for Parasitic Disease Prevention and Control, Ministry of Health, Wuxi 214064, China; 7 Jiangsu Provincial Key Laboratory on the Molecular Biology of Parasites, Jiangsu Institute of Parasitic Diseases, Wuxi 214064, China; 8 School of Data Science, Fudan University, Shanghai 200433, China; wangyaoqian@fudan.edu.cn (Y.W.); fu@fudan.edu.cn (B.F.); 9 NHC Key Laboratory of Parasite and Vector Biology, National Institute of Parasitic Diseases at Chinese Center for Disease Control and Prevention (Chinese Center for Tropical Diseases Research), WHO Collaborating Centre for Tropical Diseases, Shanghai 200025, China; guosy@nipd.chinacdc.cn (S.G.); dengwp@nipd.chinacdc.cn (W.D.); lvchao@nipd.chinacdc.cn (C.L.); zhanglj@nipd.chinacdc.cn (L.Z.); xujing@nipd.chinacdc.cn (J.X.); 10 School of Economics and Management, Southeast University, Nanjing 210096, China; huhh@seu.edu.cn

**Keywords:** schistosomiasis, zoonotic disease, One Health, system dynamics modeling, multi-objective economic evaluation, multi-criteria decision analysis

## Abstract

Schistosomiasis elimination is increasingly constrained less by the technical efficacy of single interventions than by systemic dynamics in coupled human–animal–environment settings, including nonlinear feedback, spatial heterogeneity, and cross-sectoral govern frictions. We conducted a systematic methodological review (search date: 1 January 2026) across PubMed, Web of Science, Scopus, EconLit, and CNKI to identify studies that (i) addressed schistosomiasis control, (ii) used explicit system-based, causal, or network-oriented analytical structures, and (iii) incorporated economic evaluation with multi-domain outcomes. We synthesized modeling architectures, economic methods, and approaches to trade-offs and uncertainty, and applied an evidence-informed systemic causality framework to assess decision-analytic adequacy. The literature grouped into three related strands: transmission and system dynamics models that capture feedback processes and rebound risks; economic evaluations dominated by cost-effectiveness analyses; and cross-sectoral or surveillance-oriented decision models optimizing implementation under resource constraints. Across strands, elimination-stage investments such as surveillance, environmental management, and coordination exhibit strong externalities and quasi-public-good properties that are systematically undervalued in single-sector, single-metric frameworks. We argue that decision-relevant evaluation should be reframed as a multi-objective resource allocation problem that integrates systemic modeling with economic valuation, explicitly addresses uncertainty, and applies multi-criteria decision analysis to support long-horizon, cross-sectoral decision-making.

## 1. Introduction

Schistosomiasis is a prototypical environmentally mediated parasitic disease, in which transmission is generated and sustained through the interaction of human behavior, freshwater snail ecology, and broader environmental and governance contexts [1,2,3]. Unlike infections dominated by direct interpersonal contact, schistosomiasis transmission is embedded in coupled human–environment systems, where hydrological regimes, land use, climate variability, and water infrastructure shape snail habitat suitability, while livelihoods and daily activities structure exposure patterns [4]. These processes unfold across multiple temporal and spatial scales and are mediated by public health services, surveillance capacity, and local governance arrangements [5]. As a result, schistosomiasis control is not solely a biomedical task but a social–ecological governance challenge, with transmission dynamics emerging from interactions across ecological, social, and institutional subsystems.

Over the past several decades, extensive research has been devoted to understanding the transmission mechanisms of schistosomiasis and the pathways through which interventions act [6,7]. Eco-epidemiological studies, spatial epidemiology, and system dynamics (SD) modeling have been widely applied to characterize transmission patterns, identify key environmental and behavioral drivers, and assess the epidemiological impacts of control measures [8,9]. Parallel intervention-focused research has examined how chemotherapy, snail control, environmental modification, water and sanitation improvements, and agricultural mechanization influence specific components of the transmission system [9,10,11,12]. Together, this body of work has substantially advanced understanding of how schistosomiasis transmission operates and how individual interventions modify risk along the human–snail–environment interface.

In contrast, the integration of economic evaluation into these dynamic and system-oriented analyses remains limited and methodologically underdeveloped [13,14]. Although economic analyses (e.g., cost-effectiveness studies of preventive chemotherapy) have long informed schistosomiasis control policy, they are typically conducted in parallel with, rather than embedded within, eco-epidemiological and transmission models [15]. In many cases, economic components are appended ex post to epidemiological simulations, relying on simplified assumptions regarding intervention effects, static cost structures, or short evaluation horizons [16]. This weak coupling produces a mismatch between the dynamic complexity of transmission processes and the static or quasi-static nature of economic assessment, constraining the ability of economic evaluation to inform long-term, system-level decision-making.

This limitation is further amplified by the inherently multi-domain nature of schistosomiasis control under a One Health paradigm [17,18]. Effective strategies simultaneously target human infection, animal reservoirs, and multiple environmental interfaces, including water conservancy, agriculture, forestry, and land and water management [19]. Each domain operates under distinct intervention logics, accounting conventions, temporal structures, and outcome metrics. Health-sector interventions are commonly evaluated using disease-specific outcomes, whereas environmental and agricultural measures are assessed in terms of infrastructure performance, productivity, or ecological indicators. Therefore, when combined within integrated control strategies, economic evaluation requires explicit integration and nesting across domains, including the harmonization of shared parameters, treatment of interdependencies and spillover effects, and avoidance of double counting of costs and benefits across sectors.

To date, however, few studies have explicitly addressed these integrative challenges [20,21,22]. Most economic evaluations of schistosomiasis control adopt a partial perspective, selecting a limited subset of interventions or focusing on a single sectoral slice of the system. While such analyses may yield internally consistent estimates, they fail to capture the systemic sources of cost-effectiveness that arise precisely from cross-sectoral coordination, dynamic interaction, and long-term system reconfiguration. As a result, economic evaluation often remains descriptive rather than decision-oriented, offering limited guidance for prioritization, portfolio design, and resource allocation in elimination settings, where complexity is highest and marginal gains are most costly.

Recent work has increasingly recognized schistosomiasis control as a system-level problem, emphasizing feedback loops, threshold effects, and the role of implementation and governance as endogenous drivers of outcomes [23,24]. From this perspective, control and elimination are understood not merely as reductions in infection at a given point in time, but as processes of altering the underlying causal architecture of transmission—reducing exposure opportunities, strengthening surveillance and response capacity, and sustaining low transmission under environmental and socioeconomic change. This shift highlights the need for economic evaluation frameworks capable of representing dynamic interactions, multiple objectives, and cross-sectoral system transformation. However, progress along this line remains fragmented, with limited synthesis of how economic evaluation can be meaningfully aligned with systemic causal representations of schistosomiasis transmission and control.

The objective of this review is therefore to synthesize economic evaluation research on schistosomiasis from a systemic causality perspective. Rather than focusing on individual cost-effectiveness estimates, we examine how existing studies conceptualize causal structure, embed economic valuation within dynamic transmission processes, and generate evidence relevant to transmission interruption and elimination. Accordingly, the review addresses the theoretical foundations of systemic representations of schistosomiasis transmission, methodological approaches to integrated and multi-objective economic evaluation, and the implications of these approaches for policy, governance, and resource allocation in elimination settings.

To our knowledge, this is the first review to explicitly integrate systemic causal networks with multi-objective economic evaluation in the context of schistosomiasis control. By aligning dynamic causal representations of transmission with decision-oriented economic analysis, this review advances beyond prior methodological reviews that have treated epidemiological modeling and economic evaluation in parallel.

## 2. Materials and Methods

### 2.1. Literature Search Strategy

This review followed a systematic methodological review approach, in line with the PRISMA 2020 reporting guidelines (Supplementary Material S1), to identify and synthesize studies applying system-based or causal network approaches to the economic evaluation of schistosomiasis control strategies. The review focused on conceptual frameworks, modeling strategies, and economic evaluation methods rather than estimating pooled intervention effects.

A comprehensive literature search was conducted in PubMed, Web of Science, Scopus, EconLit, and CNKI. The search was executed on 1 January 2026, including all publications in English and Chinese from database inception to that date.

The search strategy was designed around four core domains: (i) schistosomiasis, (ii) system and causal modeling approaches, (iii) economic evaluation, and (iv) multi-objective or integrated perspectives. Search terms related to schistosomiasis included “schistosomiasis”, “Schistosoma”, “snail-borne disease” and “bilharzia”. The system and causal modeling domain incorporated a broad set of terms to capture diverse modeling traditions, including “system”, “network”, and “model”. Economic evaluation terms included “economic”, “cost”, “health economics”, “cost-effectiveness”, “cost-utility”, “cost-benefit” and “economic impact”. To reflect the multi-objective and integrative nature of schistosomiasis control, additional terms such as “multi-objective”, “multi-criteria”, “trade-off”, and “One Health” were included as optional modifiers. The full search strategies for each database are provided in Supplementary Material S2.

The core search logic combined the first three domains using Boolean operators (Domain 1 AND Domain 2 AND Domain 3), with the fourth domain used to refine relevance during screening rather than as a mandatory inclusion criterion. Reference lists of included studies were also manually screened to identify additional relevant publications.

### 2.2. Study Selection Criteria

Studies were eligible for inclusion if they met the following criteria:

(1) Focused on schistosomiasis control or prevention;
(2) Incorporated an explicit system-based, causal, or network-oriented analytical structure to represent relationships among epidemiological, ecological, or intervention components;
(3) Included an economic evaluation component, such as cost-effectiveness, cost–benefit, or related economic impact assessment;
(4) Reported outcomes relevant to multiple objectives, including health, ecological, economic, or governance-related dimensions.

Studies were excluded if they:

(1) Addressed schistosomiasis but lacked any economic evaluation;
(2) Relied solely on linear or descriptive statistical analyses without an explicit system or causal structure;
(3) Evaluated only a single outcome without consideration of broader system interactions;
(4) Were editorials, commentaries, conference abstracts, or policy briefs without original analytical content.

### 2.3. Screening Process and Data Synthesis

**Figure 1:**
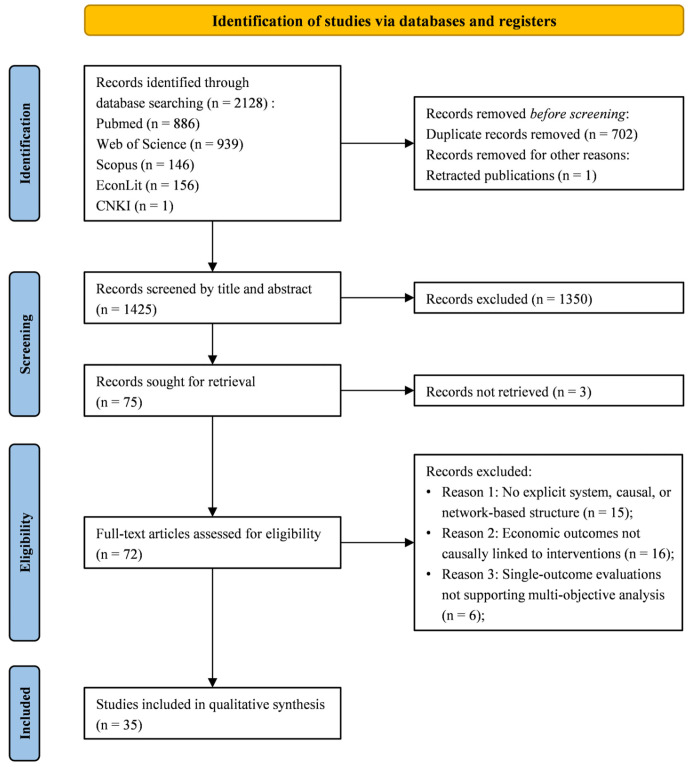
PRISMA-style flow diagram of the literature selection process.

All records retrieved from the databases were imported into EndNote (Version 21.3) for management. Duplicates were removed using a combination of automatic and manual methods: records were first deduplicated by digital object identifiers (DOIs) where available; for the remainder, EndNote’s duplicate detection function (matching on title, author, and publication year) was applied, followed by manual verification. The remaining unique records were then screened independently by two investigators in two stages: first by title and abstract, and then by full text against the eligibility criteria. Disagreements were resolved through discussion or by a third investigator. The study selection process followed a PRISMA-informed flow diagram, as illustrated in Figure 1.

### 2.4. Data Extraction and Analytical Framework

For the included studies, key information was extracted on:

(1) System or causal modeling approach;
(2) Representation of epidemiological–ecological interactions;
(3) Type and scope of economic evaluation;
(4) Treatment of multiple objectives and trade-offs.

To synthesize methodological patterns across studies, an evidence-informed network systematic diagram was constructed by identifying recurrent causal nodes and links reported in the included literature. This diagram was used as an integrative analytical tool to compare how different modeling approaches structured causal assumptions and supported multi-objective economic evaluation under varying governance contexts. Furthermore, to assess the quality and utility of the included models for decision analysis, we assessed model transparency and decision-analytic adequacy using an adapted checklist covering structural validity, calibration/validation, uncertainty characterization, and reporting completeness.

## 3. A Systemic Theoretical Framework for Schistosomiasis Control

### 3.1. Environmentally Mediated Transmission and the Systemic Production of Risk

Schistosomiasis constitutes a paradigmatic environmentally mediated parasitic disease in which transmission is not merely modulated by context, but is constituted through coupled interactions among human behavior, freshwater snail ecology, and the broader environmental–institutional milieu. This embeddedness implies that transmission risk cannot be reduced to a proximate biomedical mechanism; rather, it is endogenously produced through the co-evolution of pathogen–host–vector–environment configurations operating across multiple temporal and spatial scales.

Ecologically, the emergence, persistence, and contraction of snail habitats are continuously shaped by hydrological regimes, land-use trajectories, climatic variability, and anthropogenic ecological engineering. These drivers rarely act additively; instead, they jointly determine the spatial topology, seasonal persistence, and stability of vector populations [25]. Socially, infection risk is sustained through recurrent and frequently non-substitutable water contact associated with livelihood routines and productive activities [26]. Importantly, empirical field evidence indicates that even where mean prevalence has fallen substantially, residual transmission can be sustained by specific ecological niches and livelihood practices that preserve durable exposure channels [27].

Critically, infection is not the terminus of causation within this coupled system, rather, the disease burden propagates upstream through multiple feedback pathways—via labor capacity, household caregiving constraints, healthcare-seeking behavior, and program adherence—thereby reshaping livelihood choice sets, risk behaviors, and institutional responses [28].

Anthropogenic transformation further amplifies system dynamism by continuously reconfiguring the ecological interface. Land reclamation, wetland development, water conservancy infrastructure, and agricultural mechanization restructure aquatic landscapes and snail habitat suitability. Concurrently, population mobility and regional economic integration facilitate the propagation of residual risk beyond administrative boundaries, enabling transmission potential to be transmitted along broader mobility and hydrological networks [29]. These processes are not simply monotonic drivers of transmission intensity; they alter the architecture of exposure–contact–feedback relations, thereby reshaping the system’s spatial and causal topology [30].

Environmental pressures—climate change, hydrological volatility, ecosystem degradation, and pollution—operate through interacting and often cascading pathways. Evidence from longitudinal monitoring and cross-regional comparisons suggests that extreme precipitation can acutely expand snail distribution and exposure zones, while longer-term climatic shifts can re-delineate suitability envelopes by altering both means and variances of temperature and precipitation [31,32]. Ecosystem degradation may further erode natural buffering capacities, lowering the threshold for system perturbations to translate into transmission resurgence. Collectively, these interacting pressures render schistosomiasis transmission intrinsically dynamic and potentially unstable, particularly under conditions of compounded social–ecological change.

### 3.2. Nonlinear Dynamics and Hidden Structure in Low-Transmission Settings

A central implication of the foregoing couplings is that transmission risk does not decline proportionately with reductions in average infection prevalence—an insight that becomes especially consequential in elimination-oriented phases.

In elimination settings, an expanding literature documents that continued reductions in mean prevalence do not necessarily entail commensurate reductions in transmission potential [33]. Instead, risk becomes increasingly heterogeneous, spatially clustered, and structurally concealed. Threshold effects linking snail density, water contact patterns, and environmental disturbance imply that marginal changes in hydrology or land use can precipitate disproportionate changes in transmission intensity. Model studies have confirmed that even if the overall prevalence rate decreases, transmission can still persist through local ecological niches and highly infected subpopulations, a dynamic that is obscured by overall indicators [34]. These nonlinear dynamics are a direct consequence of risk aggregation within the system [35].

Spatially explicit analyses further indicate that residual transmission frequently aligns with hydrological connectivity, livelihood corridors, and mobility pathways, giving rise to configurations characterized by “weak links but strong feedback”—structures that are poorly detected by surveillance paradigms organized around administrative units or point-based monitoring [36]. These observations underscore a key evaluative problem, namely that single-time-point indicators and single-outcome endpoints are structurally insufficient to characterize system risk. The consequential question is not merely whether infection declines, but how intervention portfolios reshape the system’s latent risk architecture across time, space, and sectors.

### 3.3. Cross-Sectoral Governance Constraints as Endogenous Determinants

Despite the availability of technically mature tools—chemotherapy, snail control, environmental management, and health education—comparative experience consistently shows that “technical efficacy” does not automatically translate into durable system performance. The binding constraints increasingly reside in the governance system within which interventions are embedded [37].

Schistosomiasis control implicates multiple sectors (health, agriculture, water resources, environmental protection), each operating with distinct objective functions, incentive structures, and time preferences [38,39]. In the absence of credible coordination mechanisms, sectorally rational actions can aggregate into system-level suboptimality, generating offsetting or even risk-amplifying effects. The dynamic model for Schistosoma japonicum reveals that even with large-scale chemotherapy of the population, due to the lack of synchronized control of the cattle herd as the main storage host, the infection rate of the population quickly rises to the level of local epidemics after intervention is stopped, highlighting the systemic necessity of cross host prophylaxis [40].

From a systems lens, such phenomena are not implementation “noise” but structural manifestations of coordination failure in complex adaptive systems. Projects such as Geshiyaro operationalize cross-sectoral alignment between health and water, sanitation, and hygiene (WASH), probing cross-sectoral coordination mechanisms for coordinated delivery [41]. Cross-sectoral collaboration should therefore be treated as a substantive mechanism: it shapes not only immediate coverage, but also the long-run stability of elimination trajectories through learning, feedback, and path dependence. The Structure–Process–Outcome (SPO) framework reinforces that institutional arrangements do not mechanically generate effective processes; rather, process-level coordination failures systematically erode outcome robustness. In elimination contexts, the problem is intensified because benefits are distributed over time and across sectors, whereas costs are concentrated within specific departments and local jurisdictions.

### 3.4. Dynamics, Uncertainty, and the Role of Systemic Causal Networks (SCNs)

To address ecological complexity and governance constraints within a unified representation, SCNs have increasingly been adopted as an integrative backbone. In this review, SCNs are used in a narrow sense to denote explicit representations of causal structure—nodes, pathways, and feedback loops—that articulate how epidemiological, ecological, behavioral, and governance processes jointly generate system outcomes, rather than as a generic label for all system or decision-analytic models.

At the same time, many insights relevant to multi-objective economic evaluation arise from system-based transmission and decision-analytic models that do not constitute full causal networks. Models that explicitly represent cross-disease or cross-sector linkages enable economic evaluation of synergistic effects that are obscured within single-disease frameworks, particularly when benefits propagate indirectly across health domains [42].

Beyond impact estimation, system and decision-analytic modelling approaches—including transmission models and survey design models—have also been applied to the economic optimization of monitoring and evaluation strategies, supporting more efficient allocation of resources for data collection and program assessment under budget constraints [43].

Yet static causal representations are insufficient for decision analysis under elimination conditions. SD models capture accumulation, delay, rebound effects, and policy resistance—features that are central to understanding long-horizon elimination trajectories. Complementarily, Bayesian causal and hierarchical models provide principled tools for addressing data sparsity, contextual heterogeneity, and structural uncertainty in low-transmission settings, shifting inference from point estimates toward probability distributions and enabling decision-making under uncertainty [44].

### 3.5. Synthesis and the Economic Decision Problem Implied by Systemic Causality

Schistosomiasis control in elimination settings is best conceptualized not as a sequence of technical interventions, but as a long-horizon problem of steering coupled social–ecological–governance systems toward a stable low-risk attractor. Transmission risk is jointly shaped by ecological suitability, livelihood-driven exposure, environmental pressures, and coordination structures; intervention effects propagate along long causal chains with feedback and temporal delay; and performance is intrinsically heterogeneous across space and governance contexts.

Once SCNs, dynamic modeling, and uncertainty quantification are combined, the core economic question becomes how to allocate scarce resources under multiple objectives and binding governance constraints in a feedback-driven and uncertain system. The core scientific hypotheses, system characteristics, and analytical frameworks for schistosomiasis control are interrelated (Table 1).

Consequently, SCNs should be treated not as an endpoint, but as a logical starting point for multi-objective economic evaluation. The model used for economic decision-making must be able to characterize the complex interactive structures that are difficult to capture by traditional linear statistical methods and drive propagation, to ensure that the characterization of the system architecture is sufficient to support subsequent resource optimization allocation under long-term uncertainty [45].

Overall, Section 3 highlights that schistosomiasis transmission and control in elimination settings are shaped by coupled ecological, social, and governance dynamics characterized by feedback, nonlinearity, and uncertainty. These systemic features imply that economic evaluation cannot rely on static, single-sector, or short-horizon assumptions. Instead, decision-relevant analysis must be embedded within dynamic causal representations capable of capturing cross-sectoral interdependence and long-term system transformation.

## 4. The Economic Logic of Multi-Objective Schistosomiasis Control

### 4.1. Reconstructing the Economic Problem Under Elimination-Oriented Objectives

Conventional economic evaluations in morbidity-control phases typically privilege single health endpoints (e.g., prevalence reduction, cases averted, DALYs avoided). Implicitly, this paradigm assumes (i) that objectives can be proxied by a single social welfare metric and (ii) that alternatives can be compared under a common commensurate unit. Under these conditions, cost-effectiveness analysis (CEA)/cost–utility analysis (CUA) can effectively support technical choice.

Elimination-oriented control, however, fundamentally problematizes both assumptions [46]. Elimination is not the linear consequence of a single intervention but the emergent product of sustained, intersectoral investments that restructure the causal architecture of transmission over long horizons [47,48]. From an economic standpoint, the objective function expands from health maximization to the joint attainment of health, ecological integrity, economic productivity, and governance resilience—dimensions that are not independent but coupled through systemic causal pathways. The evaluative task thus shifts from “estimating health benefits” to making transparent, decision-relevant trade-offs among partially incommensurable outcomes [49].

Health outcomes capture reinfection risk and long-term morbidity; ecological outcomes include snail habitat dynamics and environmental externalities; economic outcomes encompass labor productivity, livelihood stability, and agricultural performance; and governance outcomes reflect surveillance sensitivity, response speed, coordination efficiency, and institutional robustness. Crucially, these outcomes domains are not siloed but interact systematically: ecological states shape exposure; health burdens feed back into livelihood behavior; and governance capacity modulates information asymmetries and implementation frictions. Operational modelling studies that integrate environmental components—such as snail control and xenomonitoring—within transmission modeling and cost-effectiveness analysis illustrate how adding environmental levers can reshape both epidemiological trajectories and economic efficiency relative to chemotherapy-only strategies [50].

### 4.2. Externalities and Public-Good Characteristics of Elimination-Stage Investments

The economic evaluation of elimination-stage interventions under SCNs is fundamentally challenged by their pronounced positive externalities, which extend beyond single-disease metrics to encompass broader cross-disease spillovers and long-term systemic benefits [42,51]. Environmental management yields downstream and cross-jurisdictional benefits; surveillance systems generate value primarily by reducing future outbreak probability rather than contemporaneous case counts; and WASH investments produce delayed benefits distributed across sectors and time. Similarly, vaccination programs generate herd immunity that reduces environmental transmission pressure and protect vaccinated and unvaccinated individuals in the long term but cannot be fully captured by individual level cost-effectiveness indicators [52].

The failure to internalize these cross-sectoral externalities can also generate significant negative outcomes [14]. This systematic underestimation caused by short limit horizons has been empirically demonstrated in the evaluation of schistosomiasis treatment benefits for immigrant populations [53]. These properties imply systematic underinvestment under decentralized budgeting and sector-specific performance regimes—classic welfare losses driven by uninternalized externalities rather than isolated decision errors. Moreover, many elimination-critical inputs—integrated surveillance, water safety infrastructure, ecological governance, and cross-regional coordination—approximate public or quasi-public goods. They are non-excludable (or weakly excludable) and exhibit low rivalry, with social value concentrated in maintaining a low-risk state rather than producing immediate private returns. Under the health sector evaluation framework that focuses on short-term case reduction, investments that create value by controlling and blocking environmental transmission through snail control to reduce long-term systemic risks are often systematically underestimated [54].

Balancing water infrastructure development with schistosomiasis risk minimization is achievable when projects adopt a health-sensitive design and governance approach. Rather than treating health impacts as unavoidable externalities, systemic causal modeling enables the quantification of trade-offs between infrastructure benefits, such as agricultural productivity and improved water supply, and potential health risks, including the expansion of snail habitats. This quantification allows policymakers to identify mitigation strategies, such as improved drainage design, targeted snail control, and community-based surveillance, and to integrate them into infrastructure budgets from the outset. By embedding these measures within project planning, what might otherwise be a conflict between development and disease control can be reframed as an opportunity for cross-sectoral co-benefits.

Single-sector evaluation frameworks therefore risk structurally undervaluing precisely those investments most essential to elimination sustainability. Systematic modeling studies further confirm that the strategy for eliminating schistosomiasis needs to shift from a single driver to an integrated social ecosystem [24].

### 4.3. Multi-Actor Strategic Interaction and the Economic Institutionalization of One Health

The governance of schistosomiasis elimination often resembles overlapping strategic settings: public-good provision problems with free-riding incentives, coordination games requiring credible commitment and shared information, and dynamic games in which short-term budget cycles and political incentives conflict with long-run system benefits. The central constraint is frequently incentive incompatibility: actors bearing costs are not those capturing benefits, while conventional performance metrics underweight long-run risk reduction.

This misalignment of incentives is particularly evident in practical decision-making. Empirical evidence shows that while farmers bear the costs of diagnosing and treating livestock for schistosomiasis, the associated public health benefits of reduced transmission accrue primarily to the health sector [55]. Similarly, intervention strategies organized strictly according to administrative boundaries have been proven to lead to significant resource misallocation, while using ecological zones as intervention units can improve cost-effectiveness and reduce coverage bias [56]. Those results provide empirical evidence for cross departmental collaborative intervention under the One Health framework.

Therefore, a sustainable elimination strategy requires a comprehensive approach to reveal the flow of costs and benefits across departments and periods. A three-pillar policy framework covered epidemiological, economic, and sociological dimensions was proposed [57]. It provides a structured approach to addressing these interdependent relationships. Within this framing, One Health is not merely a transdisciplinary aspiration but a governance–economic project, and the challenge is to align decentralized incentives with system-level welfare in the presence of spillovers, interdependence, and intertemporal trade-offs [58,59,60]. SCN-informed multi-objective economic evaluation contributes by rendering cross-sectoral and intertemporal benefit structures explicit and negotiable, thereby informing joint financing, benefit-sharing, and performance-linked institutional arrangements [61]. The above case and the three-pillar policy framework, demonstrates that monetizing the public health benefits of reducing zoonotic diseases, such as schistosomiasis, and incorporating them into the agricultural sector’s incentive system (e.g., subsidies) could significantly improve farmers’ treatment adoption rates. This alignment benefit is the key to system-wide gains and overall efficacy of schistosomiasis control.

Based on the evidence reviewed above, the following discussion moves from analytical characterization of systemic mechanisms to interpretation of their implications for policy and governance, which should be understood as context-dependent rather than universally prescriptive.

### 4.4. From Single-Metric Efficiency to Structured Social Choice

When objectives are multidimensional and only partially commensurable, the decision problem cannot be meaningfully reduced to a single efficiency statistic without substantial loss of policy-relevant information. In elimination-oriented settings, outcomes of concern extend beyond short-term reductions in infection to encompass ecological stability, economic productivity, and the durability of governance and surveillance capacity. Correspondingly, recent evaluative approaches increasingly shift attention from isolated interventions to portfolios and implementation intensities, using dynamic simulation to characterize the joint distribution of outcomes across time and space [62]. This shift is illustrated by recent advances in economic evaluation that integrate marginal benefit analysis with data-driven modeling approaches [63].

Within this framing, multi-criteria decision analysis (MCDA) is best viewed as a structured social choice framework rather than a mechanical ranking exercise. From a systemic causality perspective, evaluation criteria are most coherently derived from key outcome domains embedded in the causal architecture of transmission, including health risk, ecological states, economic performance, and governance capacity. Weighting thus reflects institutional priorities, budgetary constraints, and policy preferences that shape feasible trade-offs across sectors and time horizons, rather than purely technical judgments. Uncertainty, moreover, should be treated as intrinsic rather than residual: incorporating uncertainty into MCDA shifts the analytical focus from identifying a single optimal option to delineating portfolios that remain acceptable across plausible preference configurations and future scenarios. In this sense, MCDA complements systemic causal modeling by rendering trade-offs explicit, transparent, and amenable to deliberation under conditions of complexity. Comparative analyses demonstrate significant differences in the cost-effectiveness of large-scale schistosomiasis treatment when outcome indicators shift from infection rate reduction to infection intensity reduction, highlighting the methodological inadequacy of single-metric evaluation in complex transmission systems [64].

From an operational perspective, multi-criteria decision analysis (MCDA) provides a structured mechanism for implementing multi-objective evaluation rather than a purely conceptual framing. Within the proposed systemic causal framework, evaluation criteria are derived from the key outcome domains embedded in the causal structure of transmission and control, including health outcomes, ecological states, economic and social performance, and governance and surveillance capacity. Preferences over these objectives can be represented through explicit weighting schemes, allowing different policy priorities to be explored transparently by varying the relative importance assigned to each objective. In parallel, Pareto-based multi-objective optimization can be used to identify sets of non-dominated intervention portfolios that characterize the feasible trade-off space among competing objectives. MCDA is then applied to support comparison and selection within this Pareto-efficient set, rather than collapsing outcomes into a single efficiency metric. Under elimination-oriented conditions characterized by uncertainty, MCDA can be applied across scenarios and preference configurations to identify portfolios that remain acceptable under a range of assumptions, thereby supporting cross-sectoral prioritization, budgeting, and policy deliberation.

Taken together, the economic logic of elimination-oriented schistosomiasis control extends beyond single-metric efficiency comparisons toward structured trade-off analysis under uncertainty. Multi-objective evaluation frameworks, particularly when informed by systemic causal modeling, provide a basis for transparent prioritization, cross-sectoral coordination, and long-term resource allocation. This reframing strengthens the practical relevance of economic evaluation for policy negotiation and governance design in elimination contexts.

## 5. Transferability and Implementation Challenges

**Figure 2:**
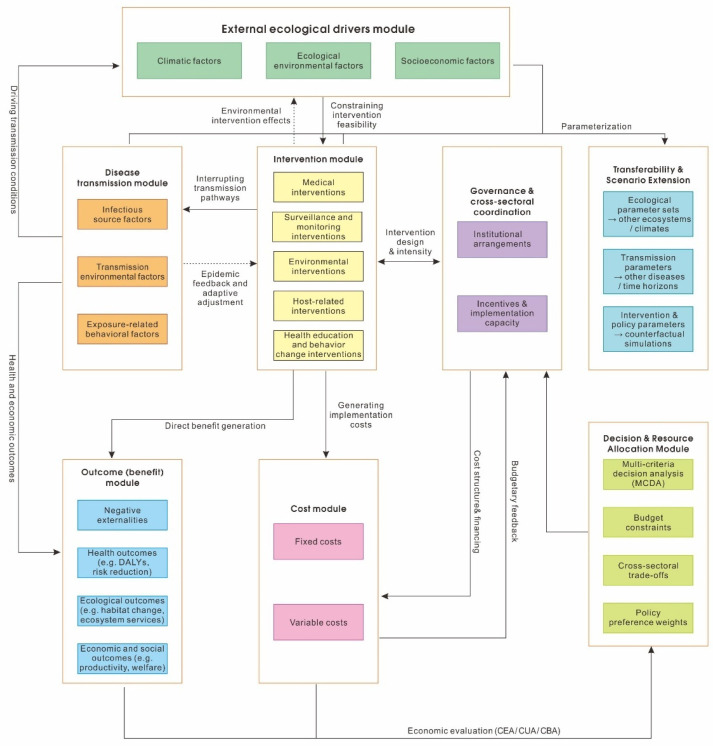
A systemic framework for multi-objective economic evaluation and resource allocation in schistosomiasis control.

Figure 2 synthesizes the systemic structure underlying the proposed economic evaluation framework, illustrating how interventions, governance arrangements, costs, and multi-dimensional outcomes are jointly integrated to inform resource allocation and decision-making under elimination-oriented conditions. It presents a systemic framework for multi-objective economic evaluation of schistosomiasis control under elimination-oriented conditions. The framework integrates disease transmission dynamics, intervention mechanisms, governance and cross-sectoral coordination, cost structures, and multi-dimensional outcomes within a unified architecture to support system-level resource allocation decisions. The implementation considerations discussed in this section are intended to illustrate how the reviewed evidence may inform policy design under different governance conditions, rather than to provide uniform recommendations.

External ecological and socioeconomic drivers—including climatic, ecological, and socioeconomic factors—shape transmission risk by influencing vector ecology, environmental exposure, and human behavior. The disease transmission module captures key components of infection risk, while the intervention module encompasses medical treatment, surveillance and monitoring, environmental management, host-related measures, and health education and behavior change interventions.

Governance and cross-sectoral coordination are explicitly modeled as endogenous system components that shape intervention design, implementation intensity, cost structures, and financing mechanisms, while also responding adaptively to implementation and budgetary feedback. Interventions generate fixed and variable costs that are distributed unevenly across sectors and time horizons, reflecting both implementation requirements and institutional arrangements.

The outcome (benefit) module captures multiple, partially incommensurable dimensions of system performance, including health outcomes (e.g., DALYs and transmission risk reduction), ecological outcomes (e.g., habitat change and ecosystem services), and economic and social outcomes (e.g., productivity and welfare), as well as negative externalities. Economic evaluation is embedded along causal pathways and time horizons rather than appended ex post, enabling identification of delayed, indirect, and cross-sectoral costs and benefits.

A dedicated decision and resource allocation module integrates conventional economic evaluation tools (cost-effectiveness, cost–utility, and cost–benefit analysis) with multi-criteria decision analysis (MCDA) to support explicit trade-offs under budget constraints, cross-sectoral spillovers, and uncertainty. Through feedback loops linking outcomes, costs, and governance, the framework conceptualizes schistosomiasis control as a dynamic, system-level resource allocation problem rather than a collection of isolated interventions.

### 5.1. Transferability as Mechanism Reconfigurability Under Scenario Uncertainty

A persistent limitation of schistosomiasis economic evaluation lies in transferability, whereby nominally similar interventions exhibit heterogeneous effects and cost structures across ecological settings and governance regimes. This heterogeneity is increasingly understood not as instability of technical efficacy, but as variation in underlying mechanism configurations and objective couplings.

Accordingly, transferability should be redefined from parameter portability to mechanism reconfigurability: whether and how the causal pathways linking interventions to multi-domain outcomes can be reconstructed under alternative ecological–social–institutional conditions. This logic has been confirmed in the prevention and control of soil borne helminth diseases: the different cost-effectiveness of large-scale drug management in Vietnam and the Philippines is due to its differentiated reconstruction of local infection structures, transmission mechanisms, and resource mobilization methods [65]. Integrating Shared Socioeconomic Pathway (SSP) narratives provides a principled way to conduct forward-looking analyses by embedding demographic change, economic development, land use, infrastructure trajectories, and governance evolution into the evaluative space [66]. Scenario analysis then serves less as prediction than as robustness testing that identifies portfolios that remain acceptable across plausible futures and thereby informing long-term planning and cross-regional collaboration [67].

### 5.2. Evidence Boundaries and Structural Tensions in Multi-Objective Evaluation

Despite conceptual appeal, multi-objective cross-sectoral evaluation faces structural tensions. First, it requires integrated evidence streams spanning surveillance, ecology, socioeconomic processes, and governance—data that are often discontinuous, non-comparable, and particularly sparse in low-transmission settings where monitoring may be deprioritized [68,69]. This data dependence and context sensitivity are particularly evident in the evaluation of screening strategies [70,71]. Beyond data availability, the fitness for purpose of diagnostic evidence itself can be a source of systematic bias [72]. Previous studies have shown that the optimal performance indicators for schistosomiasis diagnostic tools are highly dependent on the local epidemic intensity. If the diagnostic tools relied upon for evaluation do not match the local epidemiological context, it may lead to systematic decision biases (e.g., resource mismatches) [73]. Second, uncertainty is intrinsic, arising from contested trade-offs, discounting assumptions, and unknown future trajectories; deterministic rankings are therefore epistemically fragile [74]. Empirical studies have shown that systematic planning tools, such as cross departmental collaboration matrices, are crucial for transforming multi-objective evidence into coordinated actions [23]. Third, policy usability is non-trivial. Even analytically rigorous outputs may fail to influence decisions when they cannot be translated into budgetary language, performance frameworks, and planning cycles. Methodological progress must therefore be paired with institutional design that enables evaluative evidence to enter routine decision processes [75].

## 6. Conclusions

Conceptually, this review contributes to economic evaluation by recasting schistosomiasis control as a system-level welfare allocation problem characterized by externalities, public-good dynamics, and multi-objective trade-offs. The frontier of elimination-oriented schistosomiasis evaluation is shifting from isolated methodological refinement toward strengthening the overall capacity for multi-objective, cross-sectoral decision-making. This shift is less about stacking complexity and more about achieving structural congruence between evaluative frameworks and governance realities.

A key direction is modular, reconfigurable evaluation architectures that permit adaptation to contextual variation, data limitations, and evolving policy demands—avoiding one-off models and enabling iterative updating alongside program implementation. Simultaneously, the role of economic evaluation is evolving from retrospective efficiency appraisal to embedded, prospective valuation that traces costs, benefits, and risks along causal pathways and time horizons, thereby improving the justification of public-good-type investments and countering short-termism.

From a practical perspective, the integrated strategies discussed in this review can enhance cost-effectiveness in endemic areas by internalizing cross-sectoral externalities, reducing duplication of efforts, and mitigating long-term rebound risks. By coordinating interventions across health, environmental management, agriculture, and surveillance systems, integrated approaches can generate synergistic benefits that are not captured under single-sector evaluations. In elimination settings where marginal gains become increasingly costly, embedding economic evaluation within systemic causal frameworks helps identify portfolios that sustain low transmission at lower long-term social cost. Such strategies are particularly applicable in endemic regions undergoing ecological transformation or infrastructure development, where proactive coordination can prevent future transmission resurgence and avoid costly corrective interventions. At the same time, these policy-relevant insights are necessarily contingent on governance feasibility and should therefore be interpreted as analytically informed options rather than universally applicable prescriptions.

## Figures and Tables

**Table 1:** Core scientific hypotheses, system characteristics, and analytical frameworks for economic evaluation of schistosomiasis control.

Core Scientific Hypothesis	Key System Characteristics	Corresponding Analytical Framework	Methodological Rationale
Transmission risk is jointly determined by interacting social and ecological factors	Nonlinearity, multiple feedback loops, and path dependence	Systemic causal networks (SCNs)	Economic evaluation requires explicit representation of multi-factor coupling and causal structure; single-risk-factor or linear models are structurally insufficient for valuing system-level interventions
Human activities continuously reshape ecological interfaces and transmission topology	Spatial connectivity, interface amplification effects, and cross-regional diffusion	SCNs with explicit spatial structure	Costs and benefits propagate along ecological and mobility networks; administrative units or averaged indicators fail to capture residual transmission pathways relevant for economic valuation
Compounded environmental stressors generate intrinsic system instability	Threshold effects, regime shifts, and metastable states	System dynamics (SD) modeling	Intertemporal economic trade-offs depend on long-term system evolution, feedback-driven dynamics, and nonlinear state transitions that must be explicitly modeled
System-level interventions generate costs and benefits distributed unevenly across sectors and time	Externalities, intertemporal trade-offs, and public-good characteristics	Embedded cost-effectiveness, cost–utility, and cost–benefit analysis within SCN/SD models	Economic evaluation must be embedded along causal pathways and time horizons to capture delayed, indirect, and cross-sectoral costs and benefits, rather than appended ex post
Cross-sectoral coordination failures amplify implementation frictions at the system level	Misaligned objective functions, cost–benefit asymmetry, and governance feedback loops	SCNs with endogenous governance and coordination nodes	Coordination variables must be endogenized as economic determinants; otherwise, the social value of system-level and preventive investments is systematically underestimated
Coordination mechanisms shape the long-term robustness and cost-effectiveness of elimination strategies	Cross-sectoral spillovers and limited internalization of long-term benefits	Integrated SCN-SD frameworks with governance feedback loops	Comparative evaluation of long-term economic performance under alternative coordination structures is required to assess sustainability and robustness
Elimination-oriented decision-making requires explicit trade-offs among multiple, partially incommensurable objectives	Multi-dimensional outcomes, preference heterogeneity, and decision-making under uncertainty	Multi-criteria decision analysis (MCDA) integrated with SCN/SD outputs	No single efficiency metric can capture joint health, ecological, economic, and governance outcomes; MCDA operationalizes welfare trade-offs when full monetization is infeasible
Elimination settings are characterized by pervasive uncertainty	Data sparsity, contextual heterogeneity, and incomplete evidence	Bayesian causal and hierarchical models	Economic decision-making must be based on probability distributions and explicit uncertainty characterization rather than point estimates

## Data Availability

No new data were created or analyzed in this study.

## Supplementary Materials

The following supporting information can be downloaded at: https://www.mdpi.com/article/10.3390/tropicalmed11030072/s1. Supplementary Material S1: PRISMA-2020-Checklist; Supplementary Material S2: Details of the literature search strategies across five databases. Reference [76] is cited in the Supplementary Materials.

## References

[1] Léger E., Borlase A., Fall C.B., Diouf N.D., Diop S.D., Yasenev L., Catalano S., Thiam C.T., Ndiaye A., Emery A. (2020). Prevalence and distribution of schistosomiasis in human, livestock, and snail populations in northern Senegal: A One Health epidemiological study of a multi-host system. Lancet Planet. Health.

[2] Starkloff N.C., Angelo T., Mahalila M.P., Charles J., Kinung’hi S., Civitello D.J. (2024). Spatio-temporal variability in transmission risk of human schistosomes and animal trematodes in a seasonally desiccating East African landscape. Proc. R. Soc. B Biol. Sci..

[3] Ayob N., Burger R.P., Belelie M.D., Nkosi N.C., Havenga H., de Necker L., Cilliers D.P. (2023). Modelling the historical distribution of schistosomiasis-transmitting snails in South Africa using ecological niche models. PLoS ONE.

[4] Tabo Z., Kalinda C., Breuer L., Albrecht C. (2024). Exploring the interplay between climate change and schistosomiasis transmission dynamics. Infect. Dis. Model..

[5] Zhang X., Zimmerman A., Zhang Y., Ogbuoji O., Tang S. (2024). Rapid growth of private hospitals in China: Emerging challenges and opportunities to health sector management. Lancet Reg. Health West. Pac..

[6] Trippler L., Hattendorf J., Ali S.M., Ame S.M., Juma S., Kabole F., Knopp S. (2021). Novel tools and strategies for breaking schistosomiasis transmission: Study protocol for an intervention study. BMC Infect. Dis..

[7] Grimes J.E., Croll D., Harrison W.E., Utzinger J., Freeman M.C., Templeton M.R. (2015). The roles of water, sanitation and hygiene in reducing schistosomiasis: A review. Parasites Vectors.

[8] Kamara T., Byamukama M., Karuhanga M. (2022). Modelling the Role of Treatment, Public Health Education, and Chemical Control Strategies on Transmission Dynamics of Schistosomiasis. J. Math..

[9] Malizia V., de Vlas S.J., Roes K.C.B., Giardina F. (2024). Revisiting the impact of Schistosoma mansoni regulating mechanisms on transmission dynamics using SchiSTOP, a novel modelling framework. PLoS Negl. Trop. Dis..

[10] Sule M.N., El Lahham I., Munkombwe M.N., Nasike P., Gouvras A., Rollinson D., Mbaziira R., Kanshio C., De Leo G.A. (2025). Schistosomiasis and water resources development in Africa: A scoping review and multi-case evaluation of associated snail control. PLoS Negl. Trop. Dis..

[11] Adediran M.B., Adesida A., Ezekiel O.O., Irabor P.C., Babalola B.M., Oyeyemi O.T. (2025). From treatment to prevention: Reimagining schistosomiasis control through WASH and environmental management. Pathog. Glob. Health.

[12] Okesanya O.J., Eshun G., Ukoaka B.M., Manirambona E., Olabode O.N., Adesola R.O., Okon I.I., Jamil S., Singh A., Lucero-Prisno D.E. (2024). Water, sanitation, and hygiene (WASH) practices in Africa: Exploring the effects on public health and sustainable development plans. Trop. Med. Health.

[13] Chen X.-F., Li Q., Bergquist R., Zheng J.-X., Guo S.-Y., Lan Q.-F., He Z.-Z., Zhang L.-J., Cao C.-L., Xu J. (2025). Estimation and prediction on the economic burden of schistosomiasis in 25 endemic countries. Infect. Dis. Poverty.

[14] Rinaldo D., Perez-Saez J., Vounatsou P., Utzinger J., Arcand J.-L. (2021). The economic impact of schistosomiasis. Infect. Dis. Poverty.

[15] Uzoegbo S.C., Jackson L.J., Bloch S.C.M. (2022). A systematic review and quality appraisal of the economic evaluations of schistosomiasis interventions. PLoS Negl. Trop. Dis..

[16] Turner H.C., Stolk W.A., Solomon A.W., King J.D., Montresor A., Molyneux D.H., Toor J. (2021). Are current preventive chemotherapy strategies for controlling and eliminating neglected tropical diseases cost-effective?. BMJ Glob. Health.

[17] Guo S., Li L., Zhang L.-J., Li Y., Li S., Xu J. (2021). From the One Health Perspective: Schistosomiasis Japonica and Flooding. Pathogens.

[18] Zhang X.X., Guo X.K., Zhou X.N. (2025). One Health: A key element in the WHO Pandemic Agreement. Lancet.

[19] Hong Z., Li L., Zhang L.-J., Wang Q., Xu J., Li S., Zhou X. (2022). Elimination of Schistosomiasis Japonica in China: From the One Health Perspective. China CDC Wkly..

[20] Joo H., Maskery B.A., Alpern J.D., Weinberg M., Stauffer W.M. (2023). Cost-effectiveness of treatment strategies for populations from strongyloidiasis high-risk areas globally who will initiate corticosteroid treatment in the USA. J. Travel Med..

[21] Lo N.C., Coulibaly J.T., Bendavid E., N’Goran E.K., Utzinger J., Keiser J., Bogoch I.I., Andrews J.R. (2016). Evaluation of a Urine Pooling Strategy for the Rapid and Cost-Efficient Prevalence Classification of Schistosomiasis. PLoS Neglected Trop. Dis..

[22] Coffeng L.E., Levecke B., Hattendorf J., Walker M., Denwood M.J. (2021). Survey Design to Monitor Drug Efficacy for the Control of Soil-Transmitted Helminthiasis and Schistosomiasis. Clin. Infect. Dis..

[23] Nakagawa J., Ehrenberg J.P., Nealon J., Fürst T., Aratchige P., Gonzales G., Chanthavisouk C., Hernandez L.M., Fengthong T., Utzinger J. (2015). Towards effective prevention and control of helminth neglected tropical diseases in the Western Pacific Region through multi-disease and multi-sectoral interventions. Acta Trop..

[24] King C.H., Yoon N., Wang X., Lo N.C., Alsallaq R., Ndeffo-Mbah M., Li E., Gurarie D. (2020). Application of Schistosomiasis Consortium for Operational Research and Evaluation Study Findings to Refine Predictive Modeling of Schistosoma mansoni and Schistosoma haematobium Control in Sub-Saharan Africa. Am. J. Trop. Med. Hyg..

[25] Trinos J., Ng-Nguyen D., Coffeng L.E., Dyer C.E.F., Clarke N., Traub R., Halton K., Wiseman V., Watts C., Nery S.V. (2023). Cost and cost-effectiveness analysis of mass drug administration compared to school-based targeted preventive chemotherapy for hookworm control in Dak Lak province, Vietnam. Lancet Reg. Health West. Pac..

[26] Lund A., Sam M., Sy A.B., Sow O., Ali S., Sokolow S., Merrell S.B., Bruce J., Jouanard N., Senghor S. (2019). Unavoidable Risks: Local Perspectives on Water Contact Behavior and Implications for Schistosomiasis Control in an Agricultural Region of Northern Senegal. Am. J. Trop. Med. Hyg..

[27] Garchitorena A., Sokolow S.H., Roche B., Ngonghala C.N., Jocque M., Lund A., Barry M., Mordecai E.A., Daily G.C., Jones J.H. (2017). Disease ecology, health and the environment: A framework to account for ecological and socio-economic drivers in the control of neglected tropical diseases. Philos. Trans. R. Soc. B Biol. Sci..

[28] Du H., Zahn M., Loo S., Alleman T., Truelove S., Patenaude B., Gardner L., Papageorge N., Hill A. (2025). Improving policy design and epidemic response using integrated models of economic choice and disease dynamics with behavioral feedback. PLoS Comput. Biol..

[29] Lessani M., Li Z., Jing F., Qiao S., Zhang J., Olatosi B., Li X. (2023). Human mobility and the infectious disease transmission: A systematic review. Geo-Spat. Inf. Sci..

[30] Zhang X.X., Jin Y.Z., Lu Y.H., Huang L.L., Wu C.X., Lv S., Chen Z., Xiang H., Zhou X.N. (2023). Infectious disease control: From health security strengthening to health systems improvement at global level. Glob. Health Res. Policy.

[31] Tabo Z., Wangalwa R., Rwibutso M., Breuer L., Albrecht C. (2025). Future climate and demographic changes will almost double the risk of schistosomiasis transmission in the Lake Victoria Basin. One Health.

[32] Asare K.K., Mohammed M.-D.W., Aboagye Y.O., Arndts K., Ritter M. (2025). Impact of Climate Change on Schistosomiasis Transmission and Distribution—Scoping Review. Int. J. Environ. Res. Public Health.

[33] Wen-Yan F., Ling W., Xin W., Feng-Ning H. (2016). Grey relational analysis of environment interference factors and control measures on endemic status of schistosomiasis in Poyang Lake Eco-economic Region. Zhongguo Xue Xi Chong Bing Fang Zhi Za Zhi.

[34] Wang X., Gurarie D. (2012). Mathematical Models of Schistosomiasis Transmission, Morbidity and Control with Applications to Endemic Communities in Coastal Kenya.

[35] Truscott J.E., Turner H.C., Farrell S.H., Anderson R.M., Basanez M.G., Anderson R.M. (2016). Soil-Transmitted Helminths: Mathematical Models of Transmission, the Impact of Mass Drug Administration and Transmission Elimination Criteria. Advances in Parasitology, Vol 94: Mathematical Models for Neglected Tropical Diseases: Essential Tools for Control and Elimination, Pt B.

[36] Meckawy R., Stuckler D., Mehta A., Al-Ahdal T., Doebbeling B. (2022). Effectiveness of early warning systems in the detection of infectious diseases outbreaks: A systematic review. BMC Public Health.

[37] Michael E., Madon S. (2017). Socio-ecological dynamics and challenges to the governance of Neglected Tropical Disease control. Infect. Dis. Poverty.

[38] Zhang Q.Y., Zhang Y.Y., Liu J.S., Li X.C., Zhu Z.L., Feng X.Y., Han L.F., Zhu C.D., Tun H.M., Zong L. (2025). Integrating One Health governance in China: Assessing structural implementation and operational entry points. One Health.

[39] Li X., Zhang Y., Zhang Q., Liu J., Zhu Z., Feng X., Han L., Zhang X. (2025). Strategy and mechanism of One Health governance: Case study of China. Sci. One Health.

[40] Williams G.M., Sleigh A.C., Li Y.S., Feng Z., Davis G.M., Chen H.G., Ross A.G.P., Bergquist R., McManus D.P. (2002). Mathematical modelling of schistosomiasis japonica: Comparison of control strategies in the People’s Republic of China. Acta Trop..

[41] Mekete K., Ower A., Dunn J., Sime H., Tadesse G., Abate E., Nigussu N., Seife F., McNaughton E., Anderson R.M. (2019). The Geshiyaro Project: A study protocol for developing a scalable model of interventions for moving towards the interruption of the transmission of soil-transmitted helminths and schistosome infections in the Wolaita zone of Ethiopia. Parasites Vectors.

[42] Mbah M.L.N., Poolman E.M., Atkins K.E., Orenstein E.W., Meyers L.A., Townsend J.P., Galvani A.P. (2013). Potential Cost-Effectiveness of Schistosomiasis Treatment for Reducing HIV Transmission in Africa—The Case of Zimbabwean Women. PLoS Negl. Trop. Dis..

[43] Minnery M., Okoyo C., Morgan G., Wang A., Johnson O., Fronterre C., Montresor A., Campbell S.J., Mwandawiro C., Diggle P. (2024). Cost-effectiveness of comparative survey designs for helminth control programs: Post-hoc cost analysis and modelling of the Kenyan national school-based deworming program. PLoS Negl. Trop. Dis..

[44] Araujo Navas A.L., Soares Magalhães R.J., Osei F., Fornillos R.J.C., Leonardo L.R., Stein A. (2018). Modelling local areas of exposure to Schistosoma japonicum in a limited survey data environment. Parasites Vectors.

[45] Xu J.F., Xu J., Li S.Z., Jia T.W., Huang X.B., Zhang H.M., Chen M., Yang G.J., Gao S.J., Wang Q.Y. (2013). Transmission risks of schistosomiasis japonica: Extraction from back-propagation artificial neural network and logistic regression model. PLoS Negl. Trop. Dis..

[46] Lopez S., Majid S., Syed R., Rychtar J., Taylor D. (2024). Mathematical model of voluntary vaccination against schistosomiasis. PeerJ.

[47] Agbata E.N., Morton R.L., Bisoffi Z., Bottieau E., Greenaway C., Biggs B.A., Montero N., Tran A., Rowbotham N., Arevalo-Rodriguez I. (2018). Effectiveness of Screening and Treatment Approaches for Schistosomiasis and Strongyloidiasis in Newly-Arrived Migrants from Endemic Countries in the EU/EEA: A Systematic Review. Int. J. Environ. Res. Public Health.

[48] Welch V.A., Ghogomu E., Hossain A., Awasthi S., Bhutta Z.A., Cumberbatch C., Fletcher R., McGowan J., Krishnaratne S., Kristjansson E. (2017). Mass deworming to improve developmental health and wellbeing of children in low-income and middle-income countries: A systematic review and network meta-analysis. Lancet Glob. Health.

[49] Meginnis K., Hanley N., Mujumbusi L., Pickering L., Lamberton P.H.L. (2022). Using choice modelling to identify popular and affordable alternative interventions for schistosomiasis in Uganda. Environ. Dev. Econ..

[50] Allan F., Ame S.M., Tian-Bi Y.N.T., Hofkin B.V., Webster B.L., Diakité N.R., N’Goran E.K., Kabole F., Khamis I.S., Gouvras A.N. (2020). Snail-Related Contributions from the Schistosomiasis Consortium for Operational Research and Evaluation Program Including Xenomonitoring, Focal Mollusciciding, Biological Control, and Modeling. Am. J. Trop. Med. Hyg..

[51] Li X.C., Zhang Y.Y., Zhang Q.Y., Liu J.S., Ran J.J., Han L.F., Zhang X.X. (2024). Global burden of viral infectious diseases of poverty based on Global Burden of Diseases Study 2021. Infect. Dis. Poverty.

[52] Collyer B.S., Turner H.C., Hollingsworth T.D., Keeling M.J. (2019). Vaccination or mass drug administration against schistosomiasis: A hypothetical cost-effectiveness modelling comparison. Parasites Vectors.

[53] Zammarchi L., Botta A., Tilli M., Gobbi F., Bartoloni A., Boccalini S. (2023). Presumptive treatment or serological screening for schistosomiasis in migrants from Sub-Saharan Africa could save both lives and money for the Italian National Health System: Results of an economic evaluation. J. Travel Med..

[54] Lo N.C., Gurarie D., Yoon N., Coulibaly J.T., Bendavid E., Andrews J.R., King C.H. (2018). Impact and cost-effectiveness of snail control to achieve disease control targets for schistosomiasis. Proc. Natl. Acad. Sci. USA.

[55] Adeyemo P., Léger E., Hollenberg E., Diouf N., Sène M., Webster J.P., Häsler B. (2022). Estimating the financial impact of livestock schistosomiasis on traditional subsistence and transhumance farmers keeping cattle, sheep and goats in northern Senegal. Parasites Vectors.

[56] Cha S., Elhag M.S., Lee Y.H., Cho D.S., Ismail H., Hong S.T. (2019). Epidemiological findings and policy implications from the nationwide schistosomiasis and intestinal helminthiasis survey in Sudan. Parasites Vectors.

[57] Janoušková E., Clark J., Kajero O., Alonso S., Lamberton P.H.L., Betson M., Prada J.M. (2022). Public Health Policy Pillars for the Sustainable Elimination of Zoonotic Schistosomiasis. Front. Trop. Dis..

[58] Zhang X.X., Liu J.S., Han L.F., Simm G., Guo X.K., Zhou X.N. (2022). One Health: New evaluation framework launched. Nature.

[59] Zhang X.X., Liu J.S., Han L.F., Xia S., Li S.Z., Li O.Y., Kassegne K., Li M., Yin K., Hu Q.Q. (2022). Towards a global One Health index: A potential assessment tool for One Health performance. Infect. Dis. Poverty.

[60] Zhang Q., Liu J., Han L., Li X., Zhang C., Guo Z., Chao A., Wang C., Wan E., Chen F. (2024). How far has the globe gone in achieving One Health? Current evidence and policy implications based on global One Health index. Sci. One Health.

[61] Zhang X.X., Li X.C., Zhang Q.Y., Liu J.S., Han L.F., Lederman Z., Schurer J.M., Poeta P., Rahman M.T., Li S.Z. (2023). Tackling global health security by building an academic community for One Health action. Infect. Dis. Poverty.

[62] Spear R.C., Hubbard A., Liang S., Seto E. (2002). Disease transmission models for public health decision making: Toward an approach for designing intervention strategies for Schistosomiasis japonica. Environ. Health Perspect..

[63] Li Q., Zheng J.X., Jia T.W., Feng X.Y., Lv C., Zhang L.J., Yang G.J., Xu J., Zhou X.N. (2023). Optimized strategy for schistosomiasis elimination: Results from marginal benefit modeling. Parasites Vectors.

[64] Turner H.C., Truscott J.E., Bettis A.A., Farrell S.H., Deol A.K., Whitton J.M., Fleming F.M., Anderson R.M. (2017). Evaluating the variation in the projected benefit of community-wide mass treatment for schistosomiasis: Implications for future economic evaluations. Parasites Vectors.

[65] Trinos D., Caesar J.P. (2023). Cost, Effectiveness, and Cost-Effectiveness of Preventive Chemotherapy for Control of Soil-Transmitted Helminths in Vietnam and the Philippines.

[66] Intergovernmental Panel on Climate Change (2023). Framing, Context, and Methods. Climate Change 2021—The Physical Science Basis: Working Group I Contribution to the Sixth Assessment Report of the Intergovernmental Panel on Climate Change.

[67] Li X.C., Qian H.R., Zhang Y.Y., Zhang Q.Y., Liu J.S., Lai H.Y., Zheng W.G., Sun J., Fu B., Zhou X.N. (2024). Optimal decision-making in relieving global high temperature-related disease burden by data-driven simulation. Infect. Dis. Model..

[68] Liu J.S., Li X.C., Zhang Q.Y., Han L.F., Xia S., Kassegne K., Zhu Y.Z., Yin K., Hu Q.Q., Xiu L.S. (2023). China’s application of the One Health approach in addressing public health threats at the human-animal-environment interface: Advances and challenges. One Health.

[69] Sun Z., Zhou H., Chen F., Lu S., Liang H., Wan E., Tao Z., Zhao H., Zhou X., Yang F. (2023). Understanding the China-Tanzania Malaria Control Project: Lessons learned from a multi-stakeholder qualitative study. Front. Public Health.

[70] Lamberti O., Terris-Prestholt F., Bustinduy A.L., Bozzani F. (2024). A health decision analytical model to evaluate the cost-effectiveness of female genital schistosomiasis screening strategies: The female genital schistosomiasis SCREEN framework. Trop. Med. Int. Health.

[71] Manca F., Ciminata G., Grieve E., Reboud J., Cooper J., McIntosh E. (2024). Cost-effectiveness of sentinel screening of endemic diseases alongside malaria diagnosis: A case study in schistosomiasis. PLoS Negl. Trop. Dis..

[72] Coffeng L.E., Graham M., Browning R., Kura K., Diggle P.J., Denwood M., Medley G.F., Anderson R.M., de Vlas S.J. (2024). Improving the Cost-efficiency of Preventive Chemotherapy: Impact of New Diagnostics on Stopping Decisions for Control of Schistosomiasis. Clin. Infect. Dis..

[73] Chevalier J.M., Grantz K.H., Girdwood S., Kepha S., Ramos T., Nichols B.E., Khan S., Hingel S. (2025). The impact and cost of a new rapid diagnostic test for school-based prevalence mapping and monitoring and evaluation surveys of schistosomiasis: A modelling study. PLoS Negl. Trop. Dis..

[74] Zhang X., Zimmerman A., Lai H., Zhang Y., Tang Z., Tang S., Ogbuoji O. (2024). Differential effect of China’s Zero Markup Drug Policy on provider-induced demand in secondary and tertiary hospitals. Front. Public Health.

[75] Zhang X.X., Lederman Z., Han L.F., Schurer J.M., Xiao L.H., Zhang Z.B., Chen Q.L., Pfeiffer D., Ward M.P., Sripa B. (2024). Towards an actionable One Health approach. Infect. Dis. Poverty.

[76] Page M.J., McKenzie J.E., Bossuyt P.M., Boutron I., Hoffmann T.C., Mulrow C.D., Shamseer L., Tetzlaff J.M., Akl E.A., Brennan S.E. (2021). The PRISMA 2020 statement: An updated guideline for reporting systematic reviews. BMJ.

