# Supplementary material for: Economic Evaluation of Multi-Objective Schistosomiasis Control Through Systemic Causality: Theoretical Advances and Governance Implications
Source: Trop Med Infect Dis. 2026 Mar 5;11(3):72. doi: 10.3390/tropicalmed11030072 (PMC13030446; doi:10.3390/tropicalmed11030072)
Supplement: Supplementary file 1 [file tropicalmed-11-00072-s001.zip › Supplementary Material S2.pdf]

Supplementary Material S2

Table S1. Details of the literature search strategies across five databases.

Database	Date Searched	Search Strategy
PubMed	Jan 1, 2026	(schistosomiasis* OR Schistosoma OR "snail-borne disease" OR bilharzia) AND (economic* OR cost* OR "health economics" OR "cost-effectiveness" OR "cost-benefit" OR "cost-utility" OR "economic impact") AND (system* OR network* OR model*)
Web of Science	Jan 1, 2026	TS=((schistosomiasis* OR Schistosoma OR "snail-borne disease" OR bilharzia) AND (economic* OR cost* OR "health economics" OR cost-effectiveness OR "cost benefit" OR "cost utility" OR "economic impact") AND (system* OR network* OR model*))
Scopus	Jan 1, 2026	TITLE-ABS-KEY ((schistosomiasis* OR Schistosoma OR "snail-borne disease" OR bilharzia) AND (economic* OR cost* OR "health economics" OR "cost-effectiveness" OR "cost-benefit" OR "cost-utility" OR "economic impact") AND (system* OR network* OR model*))
EconLit	Jan 1, 2026	[All fields] (schistosomiasis* OR Schistosoma OR snail-borne disease OR bilharzia) AND (economic* OR cost* OR health economics OR cost-effectiveness OR cost-benefit OR cost-utility OR economic impact) AND (system* OR network* OR model*)
CNKI (search terms in Chinese)	Jan 1, 2026	SU = ('schistosomiasis' OR 'snail' OR 'Schistosoma japonicum' OR 'Schistosoma mansoni') AND SU = ('economic evaluation' OR 'cost-effectiveness' OR 'cost-benefit' OR 'health economics' OR 'economic impact') AND SU = ('system dynamics' OR 'Bayesian network' OR 'causal network' OR 'system model' OR 'network model')
